# Rapid Hemostatic Biomaterial from a Natural Bath Sponge Skeleton

**DOI:** 10.3390/md19040220

**Published:** 2021-04-15

**Authors:** Qinghua Wang, Jingwei Chen, Dexiang Wang, Minghui Shen, Huilong Ou, Jing Zhao, Ming Chen, Guoliang Yan, Jun Chen

**Affiliations:** 1 Department of Marine Biological Science & Technology, College of Ocean and Earth Sciences, Xiamen University, Xiamen 361102, China; wangqinghua@stu.xmu.edu.cn (Q.W.); cjw1301@163.com (J.C.); dxwang@xmu.edu.cn (D.W.); hlou@xmu.edu.cn (H.O.); sunnyzhaoj@xmu.edu.cn (J.Z.); ming.chen@xmu.edu.cn (M.C.); 2 State-Province Joint Engineering Laboratory of Marine Bioproducts and Technology, Xiamen University, Xiamen 361102, China; 3 Xiamen City Key Laboratory of Urban Sea Ecological Conservation and Restoration, Xiamen University, Xiamen 361102, China; 4 Hainan Academy of Ocean and Fisheries Sciences, Haikou 570206, China; smh112266@aliyun.com; 5 Basic Medical Department of School of Medicine, Xiamen University, Xiamen 361102, China; guoliangyan@xmu.edu.cn

**Keywords:** *Spongia officinalis*, spongin, hemostasis, platelet, biocompatibility

## Abstract

Uncontrolled bleeding is the main cause of mortality from trauma. Collagen has been developed as an important hemostatic material due to its platelet affinity function. A bath sponge skeleton is rich in collagen, also known as spongin. To understand the hemostatic effect of spongin, spongin materials, SX, SFM and SR were prepared from the bath sponge *Spongia officinalis*, and hemostatic experiments were performed. The SX, SFM and SR were significantly better than the positive control, type I collagen, in shortening the whole blood clotting time in vitro and hemostasis upon rat tail amputation. In a hemostatic experiment of rabbit common carotid artery injury, the hemostatic time and 3 h survival rate of the SFM group were 3.00 ± 1.53 min and 100%, respectively, which are significantly better than those of the commercial hemostat CELOX-A (10.33 ± 1.37 min and 67%, respectively). Additionally, the SFM showed good coagulation effects in platelet-deficient blood and defibrinated blood, while also showing good biocompatibility. Through a variety of tests, we speculated that the hemostatic activity of the SFM is mainly caused by its hyperabsorbency, high affinity to platelets and high effective concentration. Overall, the SFM and spongin derivates could be potential hemostatic agents for uncontrolled bleeding and hemorrhagic diseases caused by deficiency or dysfunction of coagulation factors.

## 1. Introduction

Uncontrolled hemorrhage is the main cause of mortality from heavy bleeding trauma, and the emergency control of bleeding can effectively increase the survival rate [1,2]. To rapidly reduce blood loss in a variety of bleeding conditions, hemostatic biomaterials that are easily manufactured, portable, usable and reasonably biocompatible have attracted considerable attention [3]. Fibrous collagen is present in the subendothelial matrix and can promote platelet adhesion, activation, and aggregation for primary hemostasis. Due to their unique physiological mechanisms, low immunogenicity, low cytotoxicity and excellent biocompatibility, a variety of collagens and their derivatives have been used as hemostatic materials for different occasions, such as microfibrous collagen hemostatic agents and bioresorbable hemostat sprays [4,5,6]. However, the collagens currently used in medical care are all derived from bovine and swine sources, and their application is restricted by the risk of viral infection, religious beliefs and customs [7]. Thus, in recent decades, marine collagen with a lower threat of infectious diseases while also being free of religious concerns (derived from fish, sponge and jellyfish) has attracted much attention in the fields of food, cosmetics and wound healing [8]. However, their application in hemostasis is still in its infancy, and only collagen from jellyfish [9] and Nile tilapia skin [10,11] has been confirmed to have hemostatic functions.

Marine sponges (Porifera) are the most primitive multicellular animals on Earth and have a unique skeleton composed of spicules or fiber spongin. Sponges that possess only fiber spongin skeletons are normally called bath sponges, most of which are species from the order Dictyoceratida (class of Demospongiae). Although the spongin skeleton is water-insoluble and exhibits strong acid, alkali or protease tolerance [12], spongin is classified as a member of the collagen family by its general amino acid pattern [13,14]. The spongin skeleton is a kind of biomaterial with a long history of application [15]. It has been widely used in bathing, cleaning, helmet padding, wound caring and so on for more than 3000 years. Recently, spongin skeletons have been successfully used as extreme biomimetic [16] and biomedical materials [17,18]—for example, it has been used for hemostatic surgery due to its structural characteristics, but further studies of its applications are scarce.

In this study, we endeavored to further develop the potential of spongin from *S. officinalis* as a hemostatic material. Water-insoluble (SX and SFM) and water-soluble (SR) spongin were prepared, and their hemostatic efficacies were investigated both in vitro and in vivo. A variety of experiments were performed to identify the basic hemostatic mechanism of spongin materials. Furthermore, biocompatibility, hemolysis, cytotoxicity and acute systemic toxicity were all estimated. We expect to develop a new spongin hemostatic agent to control emergency bleeding and improve the survival rate of patients.

## 2. Results and Discussion

Emergency control of bleeding is essential for the original survival and is also the best therapeutic regimen [1]. The process of physiological hemostasis mainly includes three processes: (1) The local damaged blood vessels and nearby small blood vessels constrict, which reduces the local blood flow. (2) Subendothelial collagen is exposed, causing a small number of platelets to adhere to subendothelial collagen. The adhered platelets further activate the signal pathway in the platelets, leading to platelet aggregation, forming a platelet hemostasis thrombus, blocking the wound, and achieving preliminary hemostasis, which is also called primary hemostasis. (3) After the blood vessel is damaged, the coagulation factors are activated in a certain order to generate thrombin. Finally, the soluble fibrinogen in the plasma is transformed into insoluble fibrin, which is interwoven into a network to strengthen the hemostasis, which is called second stage hemostasis. Due to the efficient recruitment and activation of coagulation factors, such as platelets, vWF, FXII and FIX, collagen has inspired its utilization as a topical hemostatic material [3,19]. Spongin, which is analogous to collagen type XIII and has a highly cross-linked fiber bundle structure [15,20], inspired us to explore its hemostatic features. Generally, collagen is enzyme- or acid-soluble, and its extraction protocol is well established [21]. However, owing to its strong acid and alkali tolerance [12], high temperature resistance [22] and protease survivability [23], spongin extraction is difficult. The main factors affecting the solubility of collagen are the covalent linkage and noncollagen components between collagen molecules.

Thus, to evaluate the hemostatic impact of different spongin, bath sponge *S. officinalis* was extracted by various treatment.

### 2.1. Preparation and Characterization of SX, SFM and SR

**Figure 1:**
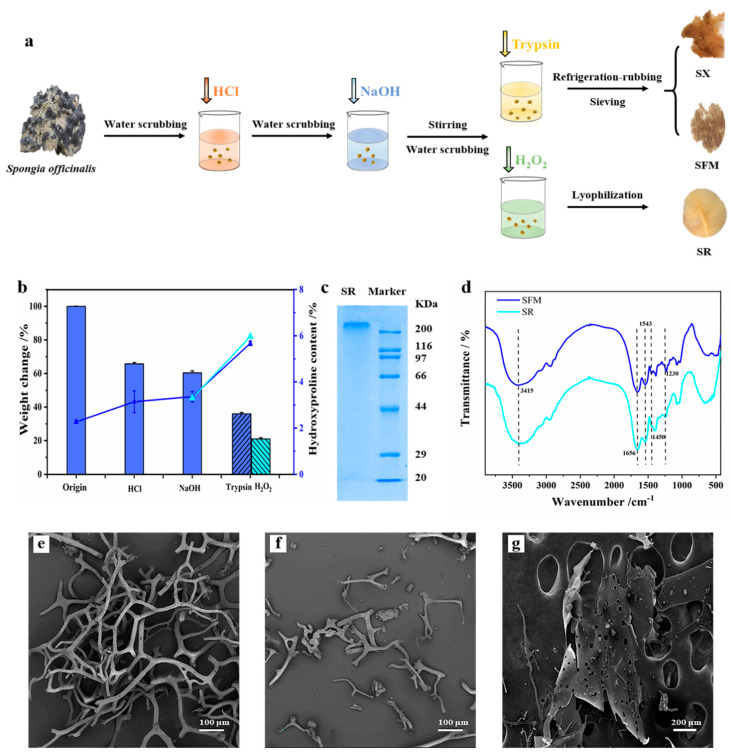
Extraction process and characteristics of spongin material. (**a**) Schematic representation showing the preparation of samples. (**b**) Sponge weight and hydroxyproline content after each step of separation and purification. Columns show changes in weight and lines show changes in hydroxyproline content. (**c**) 10% SDS polyacrylamide gel electrophoresis pattern of SR. (**d**) Fourier transform infrared spectra of the SFM and SR. SEM photographs of the SX (**e**), SFM (**f**) and SR (**g**).

*S. officinalis* skeleton was pretreated with acid and alkali to remove the impurity protein. The pretreatment of sponge skeleton was trypsin, frozen grinding and H_2_O_2_ degradation to obtain insoluble material and soluble material, respectively (Figure 1a). Collagen has unique hydroxyproline and a unique triple helix. *S. officinalis* tissue was proved to contain abundant collagen by Sirius red polarized light (Figure S1), and hydroxyproline is a unique component that is periodically distributed in the helix. Thus, the hydroxyproline concentration (Figure S2) is an index to judge the recovery and purity of spongin. After the skeleton was pretreated with acid and alkali, the weight of the water-insoluble part decreased to 60.4% of its original weight, and the hydroxyproline content increased from 2.27% to 3.40% (Figure 1b and Table S1). This result meant that approximately 90.4% of spongin still retained in the water-insoluble skeleton. A further trypsin or H_2_O_2_ treatment resulted in significant changes in the mass and hydroxyproline content. In regard to the trypsin treatment, 89.9% of the spongin was retained in the water-insoluble part with the mass changing from 60.41% to 36.0% and the hydroxyproline content changed from 3.36% to 5.67%. With regard to the H_2_O_2_ treatment, the water-soluble part (marked as SR material) was recovered with the mass changing from 60.41% to 21.0% and the hydroxyproline content changing from 3.36% to 6.03%; accordingly, the recovery rate of spongin was approximately 55.8%. According to a previous report, the hydroxyproline content in marine sponge collagen is approximately 6.0%~8.3% [13,24], and the percentage of collagen in our spongin materials was larger than 68%.

By adopting this method, we obtained insoluble rigid spongin (SX/SFM) with a hydroxyproline content of 5.67%. To make powder, which is one general form of hemostatic products, spongin was ground and separated into SX and SFM by a mesh. Clearly, there is no chemical difference between SX and SFM. In another attempt, we obtained the water-soluble material SR by a hydrogen peroxide treatment. It is surprising that the SR has a uniform protein band with a molecular weight greater than 200 kDa, as demonstrated by SDS-PAGE electrophoresis (Figure 1c). This result indicated that the molecules that made up the SR material were relatively uniform.

### 2.2. FTIR Spectra of the Spongin Materials

The FTIR spectra of the SFM and SR are shown in Figure 1d. The specific absorption bands of collagen come from amides A and B, as well as amides I, II, and III [25,26,27]. The shift of these bands generally indicates that variation occurred in the secondary structure of the sample collagen. The amide A band usually indicates the N-H stretching vibration and occurs in the range of 3440–3279 cm^−1^. If it is associated with a hydrogen bond, its absorption peak will shift to the right (redshift). The amide A band of the SFM and SR was observed at approximately 3415 cm^−1^, indicating that hydrogen bonding was weak. The amide I band normally peaks at approximately 1659 cm^−1^ and indicates the carbonyl group (C=O) stretching vibration. The band will redshift if associated with a COO^−^ group. The SFM and SR peaks are at approximately 1656 cm^−1^, indicating that their association with the COO^−^ group was weak. The peak at 1543 cm^−1^ belonged to the N-H bending vibration and C-N stretching vibration of the amide II band, reflecting the secondary structure of an α helix [14]. The peak at approximately 1230 cm^−1^ belonged to the amide III band and was evidence of the existence of a helix structure. If the absorption intensity ratio of A_1230_/A_1450_ was near 1, then the helix structure was preserved very well [28]. In regard to the SFM and SR, the ratios were approximately 1.03 and 1.37, respectively. This result indicated that the helix structure was very well preserved in the SFM but might unwind during the extraction of the SR.

The FTIR spectrum shows that the helix structure of the SR is somewhat unwound after the H_2_O_2_ treatment and heating. This result reminds us of a collagen-derived material called gelatin, which is formed from the denaturation or hydrolysis of collagen and can partly reassemble into a gel with a molecular weight of approximately 150 kDa [3]. However, whether the SR material is analogous to gelatin requires more evidence.

### 2.3. Morphology, Fluid Absorption Capacity and Porosity

Figure 1e–g and Figure S3 show the microstructure of the spongin materials, and Table S2 shows the FAC and porosity features. The SX had a mesh-like structure with an FAC of 40.5 times its own weight and a porosity of 75.4%. The SFM had a branching structure with an FAC of 994.3% and a porosity of 90.46%. It was obvious that the decrease in the FAC of the SFM material was caused by the loss of a rigid three-dimensional mesh structure after grinding the skeleton into small particles. The SR had a large number of pores on its surface, with a FAC of 362% and a porosity of 86.67%. Furthermore, spongin materials have higher FAC and porosity values than type I collagen.

### 2.4. Hemostatic Performance In Vitro

#### 2.4.1. Clotting Performance on Whole Blood

**Figure 2:**
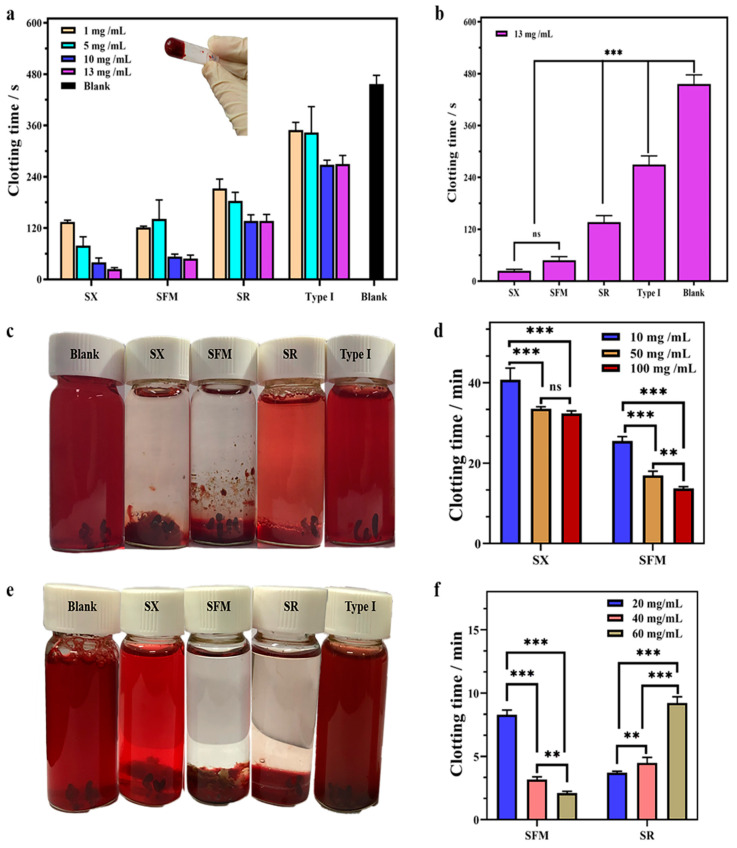
In vitro blood coagulation. (**a**) The effects of contents of the SX, SFM, SR, and type I collagen on whole blood clotting. (**b**) Clotting time of 13 mg/mL SX, SFM, SR and type I collagen to whole blood. (**c**) Photographs of the coagulation effect of blank, SX, SFM, SR and type I collagen in PDB. (**d**) The effects of contents of the SX and SFM on PDB clotting. (**e**) Photographs of the coagulation effect of blank, SX, SFM, SR and type I collagen in DSB. (**f**) The effects of contents of the SFM and SR on DSB clotting. ** *p* < 0.01 and *** *p* < 0.001.

To detect the hemostatic effect of spongin in vitro, recalcification clotting measurements were performed. As shown in Figure 2a,b and Table S3, the spongin material had an obvious coagulation effect on whole blood compared with the blank. With increasing concentrations of the SX, SFM, SR and type I collagen, the clotting time was generally shortened. When the content was nearly 13 mg/mL, the clotting times of the SX, SFM and SR were observed to be approximately 24 ± 4 s, 48 ± 8 s and 136 ± 15 s, respectively, significantly shorter than that of type I collagen (275 ± 23 s). The natural recalcification time was approximately 456 ± 20 s in the control group. In other words, compared to the blank, the 13 mg/mL doses of the SX, SFM and SR shortened the clotting time of whole blood by 88.4%, 86.8% and 70.2%, respectively. These data were significantly (*p* < 0.01) better than the 39.7% of the positive control (type I collagen).

#### 2.4.2. Clotting Performance in PDB

In regard to PDB clotting, the SX and SFM materials showed significant coagulation effects compared with the SR and type I collagen (Figure 2c,d and Figure S4, and Table S4). The SX (50 mg/mL) promoted PDB to complete coagulation in approximately 30 min, but the coagulation effect was not sufficient and some RBCs ruptured. In contrast, 50 mg/mL SFM had a more prominent coagulation effect on PDB, and the complete clotting time was approximately 17 min. Moreover, the increasing concentration of the SFM clearly shortened the clotting time. Different from the above two materials, the SR demonstrated difficulty in completely clotting, and it was observed that the blood at the top remained flowing. Even when increasing the amount of the SR, PDB was uncoagulated. The blank and type I collagen groups failed to coagulate PDB after 60 min of incubation.

#### 2.4.3. Clotting Performance in DSB

The defibrinated blood clotting test detected that the SFM and SR materials had a significant coagulation effect on DSB (Figure 2e,f and Figure S5, and Table S5). The blank, type I collagen and the SX exhibited no coagulation in the defibrinated blood after incubation for 1 h. The SFM and SR could complete the coagulation reaction, but there were differences in coagulation time and dose requirements. It was clear that the clotting effect of the SFM on DSB was dose-dependent. By increasing the amount of the SFM from 20 to 60 mg/mL, the coagulation time was effectively reduced from approximately 8.3 to 2.1 min. The SR coagulation effect showed no dose-dependent pattern. A low dose (10 mg/mL) of the SR could effectively complete the overall coagulation. However, increasing the SR dose did not significantly improve coagulation.

### 2.5. Hemostatic Performance In Vivo

**Figure 3:**
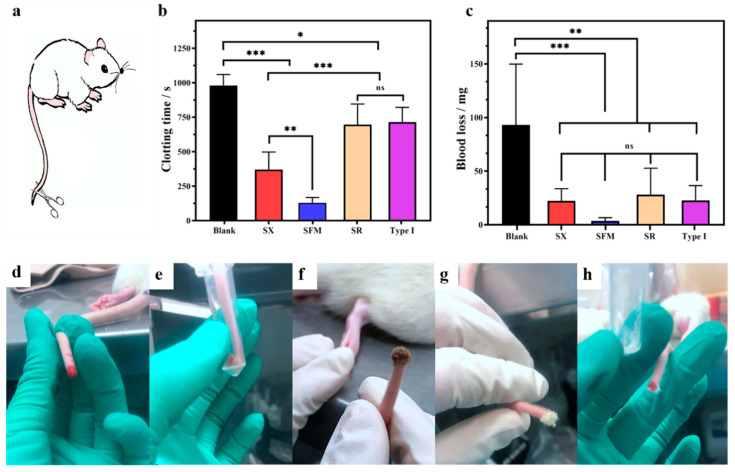
Hemostasis in rat tail amputation. (**a**) Schematic diagram of rat tail amputation. (**b**) Hemostatic time and (**c**) blood loss in the rat tail amputation model. (**d**–**h**) Picture of hemostasis with each material ((**d**): blank; (**e**): SX; (**f**): SFM; (**g**): SR; and (**h**): type I collagen). * *p* < 0.05, ** *p* < 0.01 and *** *p* < 0.001.

Rat tail amputation was conducted to evaluate in vivo hemostatic performance (Figure 3a and Table S6). Bleeding ceased in the blank group at approximately 15 min. The hemostatic time and blood loss of the test materials were clearly decreased. The hemostatic time and blood loss of the SX were 371 ± 127 s and 22.3 ± 11 mg, respectively, and those of the SR and type I collagen were roughly equivalent (nearly 705 s and 25 mg, respectively). Significantly, the SFM showed even smaller values of 129 ± 39 s and 3.6 ± 3 mg. Compared to the untreated group, treatment with the SX, SFM, SR and type I collagen shortened the in vivo hemostatic time by approximately 59%, 87%, 22% and 21% (Figure 3b) and reduced blood loss by approximately 76%, 96%, 71% and 74% (Figure 3c), respectively. It is worth noting, as shown in Figure 3d–h, that the SFM, SR and type I collagen could rapidly and fully stick to a bleeding wound. However, the SX material was fluffy and easily fell off the wound, thereby requiring continuous external pressure to maintain contact with the wound; thus, its hemostatic performance was significantly weaker than that of the SFM material.

**Figure 4:**
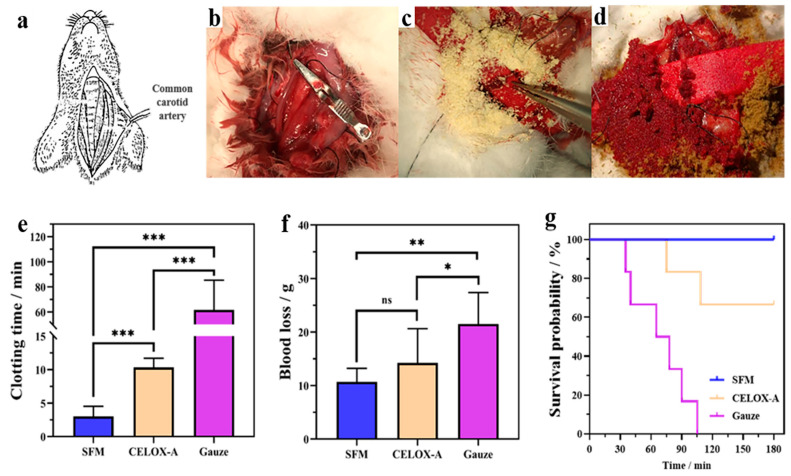
Hemostasis in rabbit′s common carotid artery incision. (**a**) Schematic diagram of New Zealand White Rabbit. (**b**) Exposing common carotid artery. (**c**) CELOX-A stopped the bleeding. (**d**) SFM stopped the bleeding. (**e**) Hemostatic time after treated with different samples. (**f**) Blood loss after treated with different samples. (**g**) Three-hour survival curve. * *p* < 0.05, ** *p* < 0.01 and *** *p* < 0.001.

Arterial bleeding is an acute form of bleeding with a high mortality rate. The rabbit common carotid artery injury model was created to evaluate hemostatic efficacy of spongin materials (Figure 4a,b). CELOX-A hemostatic (MedTrade Products Ltd., Crewe, UK) is a kind of chitosan powder that provides a high contact surface area for interaction with blood and has been widely applied in the hemostasis treatment of severe bleeding wounds in prehospital, hospital and battlefield scenarios [3,29]. Thus, in this experiment, CELOX-A was used as a positive control. Medical gauze and 100 g pressure alone did not stop the bleeding at all, and all of the rabbits in the group were died within 2 h after treatment. Both the SFM and CELOX-A were successful in hemostasis (Figure 4c,d). The hemostatic time of the SFM (3.00 ± 1.53 min) was high significantly (*p* < 0.001) shorter than that of CELOX-A (10.33 ± 1.37 min) (Figure 4e). As shown in Figure 4f, blood loss in the SFM group (10.70 ± 2.52 g) and the CELOX group (14.23 ± 6.40 g) were significantly lower than the standard gauze group (21.5 ± 5.89 g). Moreover, the survival rate of the SFM group (100%) was higher than that of CELOX group (67%) and gauze group (0%) (Figure 4g), suggesting that the hemostatic effect of the SFM was better than that of CELOX-A hemostatic powder and standard gauze to avoid excessive bleeding and death. The SFM showed better hemostatic effect than CELOX-A in the rabbit arterial bleeding model, indicating that the SFM material is worthy of further mechanism investigation and product development.

### 2.6. Hemostatic Mechanism of SFM

The hemostatic results showed that the SFM material not only efficiently accelerated the coagulation of whole blood, platelet-deficient blood and defibrinated blood in vitro, but also efficiently accelerated hemostasis in vivo. To understand the hemostatic mechanisms of the SFM material, APTT and PT measurements and platelet adhesion and RBC adhesion experiments were carried out.

#### 2.6.1. APTT and PT Test

The APTT and PT results of SFM and type I collagen were significantly decreased compared with those of the blank group (*p* < 0.01) (Table S7), indicating that both materials acted as exogenous coagulation elements that promoted clotting by activating endogenous and exogenous coagulation elements in the blood.

#### 2.6.2. Platelet, RBC and Fibrin Affinity

**Figure 5:**
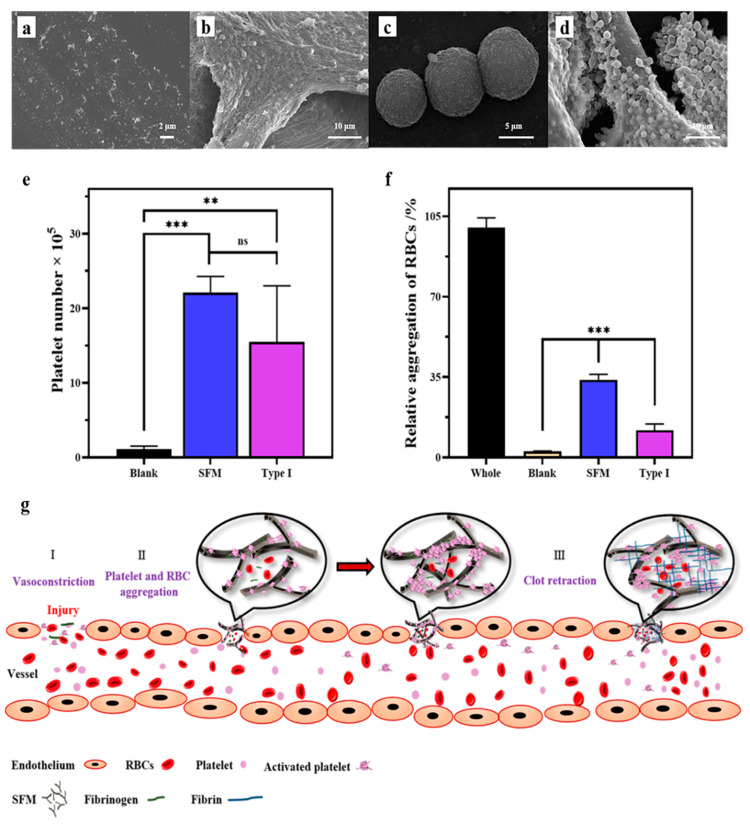
Platelets and RBCs adhered to the surface of the SFM. (**a**) Primitive morphology of platelets. (**b**) Platelets adhered to and aggregated on the SFM. (**c**) Original morphology of RBCs. (**d**) RBCs adhered to and aggregated on the SFM. (**e**) Number of platelets adhered to SFM and type I collagen after incubated for 15 min in PRP; (**f**) RBCs aggregation on SFM and type I collagen after incubation for 10 min. ** *p* < 0.01 and *** *p* < 0.001. (**g**) Schematic illustration showing the potential hemostatic mechanisms of SFM.

Changes in platelets and RBCs before and after contact with the SFM are shown in Figure 5. The inactive platelets were irregular granules (Figure 5a), and normal RBCs in the experiment were round (Figure 5c). The SFM induced a large number of platelets to aggregate, which made highly differentiated platelets form cross-linked bodies (Figure 5b and Figure S6d–f). These cross-linked bodies formed a three-dimensional fishnet structure, which could effectively intercept and recruit free red blood cells to form a complete blood clot. The quantification of platelet adhesion was achieved by the modified lactate dehydrogenase (LDH) method [30]. After the initial 15 min of incubation, the number of adhered platelets on SFM and type I collagen was significantly higher than those in the blank group (Figure 5e).

Interestingly, it was observed that the microstructure of the blood clot formed in DSB by SFM (Figure S7) was highly similar to the platelet clot around SFM, implying the defibrinated blood clot was most likely formed by the cross-linking of platelets. The SFM also showed good affinity to RBCs (Figure 5d and Figure S8f–j). The RBCs basically maintained a healthy and intact morphology after contact with SFM, which induced the RBCs to extend out microfilaments and pseudopodia. Furthermore, the quantification of RBC adhesion was detected by measuring the content of hemoglobin released from the attached RBCs. Compared with the blank group, The SFM and type I collagen both showed significant affinity to RBCs. While the relative RBC absorption of the SFM was 33.8% after incubation for 10 min, which outclassed that of type I collagen (11.8%, Figure 5f). However, the RBCs–SFM aggregation was incompact and no obvious fishnet microstructure could be observed (Figure S8g–i).

The PDB–SFM clot (Figure S9) showed different a micromorphology compared to platelets–SFM clot (Figure S6d–f), DSB–SFM clot (Figure S7) and RBCs–SFM aggregation (Figure S8f–j). The most unique microstructure of the PDB–SFM clot was the membrane that covered all the surface of the SFM (Figure S9b), which showed a dense network microstructure (Figure S9d). Compared to the DSB blood and RBC solution, the most unique blood coagulation factor in PDB was fibrin, and the membrane was most likely formed by cross-linking of fibrin. This implies that the SFM also has an affinity to fibrin.

Summarizing all the above data, we speculated that the main hemostatic mechanism of spongin, especially SFM, involved three parameters—i.e., its liquid absorption, affinity to platelets, RBCs and fibrin, and effective concentration. First, SX, SFM and SR all have ideal water-absorbing properties, which can speed up the interaction between a material and blood at the wound site when the material is applied. Figure 4 shows that, compared to CELOX-A (Figure 4c), the SFM material has excellent wettability to blood (Figure 4d). This function is similar to cotton, cellulose or their derivatives, in which they can absorb a large volume of blood and are thought to trigger hemostasis due to the material surface polarity [31,32]. Certainly, the surface physical and chemical characteristics of spongin, such as its hydrophilicity, surface charge and surface energy, require further investigation.

Second, spongin, especially SFM, has an excellent affinity for platelets, RBCs and probably fibrin (Figure 5 and Figures S6–S9). Platelet adhesion to collagen and other extracellular matrix constituents of the vessel wall and tissues is generally viewed as the first step of hemostasis [33]. Collagen can bind to platelets via the platelet surface GPIa/IIa and GPVI receptor proteins [34]. Collagen can also directly bind other coagulation factors, such as FXII and FIX [35]. The chemical similarity between spongin and collagen suggests that spongin may recruit coagulation factors with a similar mechanism. The APTT and PT tests confirm that the SFM acts as an exogenous element that triggers the endogenous and exogenous coagulation pathway. However, the detailed characteristics that enable SFM to be more effective than collagen require further study. Affinity to RBCs and fibrin of SFM may also contribute the hemostatic efficacy.

Finally, we speculate that the SFM has a higher effective concentration than the SX. The in vitro whole blood clotting experiment shows that the clotting time of the SX and SFM is dose-dependent (Figure 2a). This result means that the hemostatic effect of the SX/SFM is proportional to their effective concentration in the blood. The only difference between SX and SFM is that the SX has a rigid three-dimensional mesh structure, which is destroyed in the SFM. Such structural changes can result in changes in the effective concentration on two occasions. The first occasion comes at the very beginning of the material being applied to the bleeding wound. At this moment, the SFM can rapidly and fully stick to the blood, while the SX is fluffy and needs more time or external pressure to fully contact the blood. As a consequence, the SFM has a higher effective concentration and can trigger clotting reactions faster at this moment. This observation can explain why the SFM has better hemostatic performance in vivo than the SX. The second occasion comes at the stage in which fibrin grows into a network. Figure 2e,f shows that the SFM can clot defibrinated blood and that this function is dose-dependent. In the normal hemostatic process, soluble fibrinogen is activated by the coagulation cascade and transforms into insoluble fibrin, which further grows into a clot network [33]. This network cannot be constructed when fibrinogen or fibrin is absent. To explain why SX cannot clot DSBs at the same dose of SFM, we propose the following assumption. The SFM is fragmented, and in some locations, it has a high concentration—i.e., the distances among fragments are very small. After adhering a large number of platelets and blood cells, the SFM fragments are close enough to connect with each other by the presence of more platelets and blood cells, thereby forming a clot network. Although the SX fibers also adhere to the same number of platelets and blood cells, their rigid structure inhibits fibers from getting close and makes the clot network difficult to form. More experiments are required to confirm this assumption.

Thus, we propose the potential hemostatic mechanisms of SFM, as demonstrated in Figure 5g. Initially, the SFM is applied to the bleeding wound, and it rapidly and fully adheres to the blood before beginning to recruit platelets and other coagulation factors. As platelets attach to SFM and each other, they become activated and release a variety of chemicals to promote platelet aggregation. Then, the coagulation cascade is activated and magnified. Soluble fibrinogen is transformed into insoluble fibrin and further grows into a network, which traps blood cells and additional platelets to form a blood clot. In the meantime, the SFM fragments, which adhere to a large number of platelets and blood cells, interact either with each other or with fibrin to speed up the growth of the blood clot. Finally, the clot seals off the damaged portion of the vessel, and the wound enters the healing phase.

### 2.7. Biocompatibility of SFM

**Figure 6:**
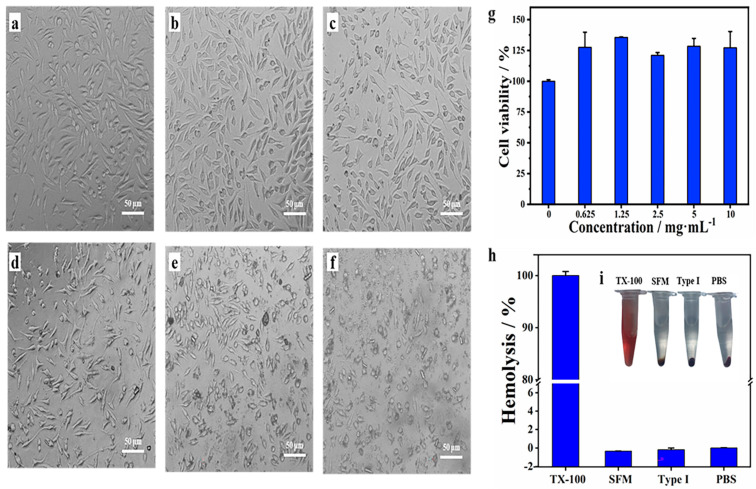
Biocompatibility of the SFM. (**a**–**f**) Image of L929 cells in 96-well plates cultured in the media with 0, 0.625, 1.25, 2.5, 5 and 10 mg/mL SFM, respectively. (**g**) Cytotoxic effects of the control and different doses of SFM after 24 h. Data represent the mean ± SD (*n* = 3). (**h**) Hemolytic percentage of the sample. (**i**) Photographs from hemolytic activity assay of the sample using PBS as negative control and TX-100 as positive control. (TX-100: 0.1% triton-X100; Type I: type I; collagen; PBS: phosphate buffered saline)**.**

Hemostatic material should not only have a good hemostatic effect but also have biocompatibility. So, the hemolytic potential, cytotoxicity, and acute systemic toxicity were evaluated. As demonstrated in Figure 6a–g, L929 cells showed over 100% viability at different doses (0.625–10 mg/mL) of the SFM and after culturing for 24 h. Thus, it was evident that the SFM had no obvious cytotoxicity to L929 cells. Consistent with previous reports, sponge protein is considered to be a promising biomaterial for bone tissue engineering [18,36], supporting the growth of human osteoprogenitor cells [17] and osteoblast-like MG-63 cells [37]. In the hemolysis test, no hemolysis was observed in the SFM, type I collagen or PBS. After 1 h of incubation at 37 °C, erythrocyte sedimentation occurred in all the above-mentioned samples, and the upper liquid was colorless (Figure 6h,i). Finally, rats injected with SFM extract showed no signs of toxicity, such as dyspnea, decreased activity, vomiting, diarrhea, convulsions or gait imbalance (Table S8). The body weight change after 72 h also showed no difference between the SFM group and blank group (*p* > 0.05). Consistent with these reports, our results show that the SFM has good biocompatibility. All in all, the SFM has great development potential due to its promising hemostatic effect and good biocompatibility.

## 3. Materials and Methods

### 3.1. Materials and Methods

*S. officinalis* was collected in the intertidal zone of Danzhou, Hainan Province, China. Chemical reagents were purchased from Sinopharm Chemical Reagent Co., Ltd. (Shanghai, China). Bovine type I collagen powder was acquired from Solarbio Life Sciences Co., Ltd. (Beijing, China). CELOX-A, a chitosan powder, was provided from Medtrade Products Ltd. (Electra House, Crewe Business Park, Crewe, CW1 6GL, UK). Prothrombin time (PT) assay kit (Y201) and activated partial thromboplastin time (APTT) assay kit (Y217) were obtained from Shanghai Sun Biotechnology Co., Ltd. (Shanghai, China). Citrated rabbit whole blood was got from Ruite Biotechnology Co., Ltd. (Guangzhou, China). Defibrinated sheep blood (DSB) came from Maojie Microorganism Technology Co., Ltd. (Nanjing, China). Male Sprague–Dawley (SD) rats (0.2–0.3 kg) were provided by the Animal Experiment Center in Medical College of Xiamen University, China (Experimental Animal Production License: SCXK (Min) 2018-0003). Male New Zealand white rabbits (1.9 ± 0.4 Kg) were obtained from Songlian Experimental Animal Farm of Songjiang District, China (Experimental Animal Production License: 20170008000817). Under the Ethical approval number XMULAC 20200044, both rat and rabbit experiments were performed by following the animal protocol which was approved by the Xiamen University Animal Ethical Committee.

### 3.2. Preparation of SX, SFM and SR 

#### 3.2.1. Pretreatment of Sponge Skeleton

Through rubbing and washing, cells of *S. officinalis* were removed and sponge skeleton was obtained. Then, the resulting skeleton was cut into small pieces and continuously immersed in 0.8 M HCl solution (24 h) and 0.1 M NaOH solution (24 h) at room temperature. After each procedure, it was washed in neutral by ultrapure water.

#### 3.2.2. Preparation of SFM and SX

The pretreated sponge skeleton was dispersed in 0.1% trypsin (pig pancreas, 1:250) with 0.1 M phosphate buffered saline (PBS, pH 7.8) and stirred for 24 h. Then, the treated skeleton was washed by ultrapure water and dried. Finally, it was grinded by a freezing grinder (JXFSTPRP-ІІ-01, 15 min, −10 °C) and sifted by a mesh with pore size of 74 μm. The part that sifted through the mesh was named SFM, and the part retained on the mesh was named SX.

#### 3.2.3. Preparation of SR 

The degraded sponge skeleton was dispersed in a 10% H_2_O_2_ solution (1:10, V/V) and stirred at 55 °C for 4 h. The suspension was centrifuged (1700× *g*, 30 min), and the supernatant was dialyzed in pure water at 4 °C for 4 d and lyophilized to obtain a water-soluble powder, named SR.

### 3.3. Characterization 

#### 3.3.1. Hydroxyproline Content Detection

The hydroxyproline content of the samples was assayed through the use of hydroxyproline assay kits (A030-2-1, Nanjing Jiancheng Bioengineering Institute, Nanjing, China) by following the instructions of the manufacturer.

#### 3.3.2. Microscope Analysis

The morphology was observed with an inverted microscope (Olympus, CKX41, Co. Ltd., Tokyo, Japan). The microstructure was observed by using scanning electron microscopy (SEM, JEOL JSM-6390 LV, Tokyo, Japan). The sample was fixed overnight with 2.5% glutaraldehyde at 4 °C and rinsed with 0.1 M PBS 3 times. Then, gradient dehydration was carried out using 30%, 50%, 70%, 90%, and 100% ethanol for 15 min each time. The sample was dried, coated with gold and observed by SEM.

#### 3.3.3. Fourier Transform Infrared (FTIR) Spectroscopy Characterization

The sample and potassium bromide (KBr) were dried in an oven at 60 °C to a constant weight and mixed at a ratio of 1:50. The mixture was ground to powder and pressed into a thin slice tablet by a pressing mold. The tablet was placed in the sample chamber of the Fourier transform infrared spectrometer (Nicolet IR 200, Thermo Nicolet Corp., Madison, WI, USA) and was scanned by infrared light over a wavelength range of 4000–400 cm^−1^ and a resolution of 4 cm^−1^. Each sample was scanned 32 times.

#### 3.3.4. Fluid Absorption Capacity (FAC) Measurement

The fluid absorption ratio of the materials was determined in simulated body fluid (SBF, pH 7.4) by calculating the maximum quantity of fluid absorption per unit weight of dried samples [30]. The FAC was obtained by the following formula:

(1) $$\;\mathrm{FAC}(\%)=\frac{F_{t}\;{-\;F}_{0}}{F_{0}}\;\times \;100\;$$

where F_0_ represents the weight of the dry material and F_t_ represents the weight of the wet material, which was prepared as SBF being added dropwise to the dry material until the surface leaked out excess water that was removed by filter paper. Type I collagen was selected as the control group. All experiments were conducted in triplicate with the samples.

#### 3.3.5. Porosity Measurement

The sample was dried in an oven at 60 °C to a constant weight (W_s_) and then transferred to a clean and dried density bottle. A portion of ethanol was added to the bottle and ultrasonically shaken to remove air bubbles. After the density bottle cooled to room temperature, ethanol was added to reach the marker line, and the bottle was weighed (W_a_). Then, the sample was carefully removed, and the mass of the density bottle with the remaining ethanol was weighed (W_b_). Next, ethanol was added again to the marker line, and the bottle was weighed (W_w_). The porosity was calculated according to the formula:

(2) $$\mathrm{Porosity}\;\left(\%\right)=\frac{W_{a}-{\;W}_{s}\;{-\;W}_{b}}{W_{w}\;{-\;W}_{b}}\;\times \;100\;$$

### 3.4. Hemostatic Properties

#### 3.4.1. Preparation of Platelet-Deficient Blood (PDB)

Fresh citrated rabbit whole blood was centrifuged at 200× *g* for 15 min to separate the upper layer of plasma and lower layer of red blood cells. Afterward, the plasma was centrifuged at 1200× *g* for 15 min to acquire the upper three-quarters of the platelet-poor plasma (PPP) and the bottom quarter of the platelet-rich plasma (PRP). Finally, the PPP and red blood cells were mixed in equal proportions to obtain PDB.

#### 3.4.2. Hemostasis In Vitro

Two milliliters of blood (whole blood, PDB or DSB) was mixed with samples by rapidly vibrating for a few seconds in a polypropylene tube. Afterward, 300 μL of 0.025 M CaCl_2_ was added and mixed to start coagulation. The tube was incubated at 37 °C and the clotting time was measured by tilting the tube 45° every few seconds until firm clotting was detected. The blank and type I collagen were used as the controls, and each group was tested in triplicate.

To visually demonstrate the coagulation effect, another set of coagulation experiments on the PDB and DSB blood was conducted in glass bottles. At the end of the experiment, pure water was added to lyse the free red blood cells. The degree of pigment release is generally inversely proportional to the clotting degree of blood clots.

#### 3.4.3. Rat Tail Amputation

Rat tail amputation was carried out as described by Jin′s work [38]. Rats were anesthetized by intraperitoneal injection with 10% chloral hydrate (0.03 mL/kg). One centimeter of the tail tip was cut off by surgical scissors, and the remaining tail kept naturally bleeding for 15 s before the test material was used to fully cover the wound with minimal pressure. The hemostatic time and blood loss were recorded. Five rats were assessed for each sample. The blank and type I collagen were used as the controls. All manipulations were completed under aseptic conditions. At the end of the last step, the animals were euthanized humanely with an intravenous overdose of pentobarbital sodium. Each group consisted of 5 parallel experiments.

#### 3.4.4. Rabbit′s Common Carotid Artery Incision

The rabbits were anesthetized with 20% ethyl carbamate (5 mL/Kg) in the ear margin vein and fixed on the experimental table. The neck was clipped and the neck skin (5–7 cm) was cut close to the lower margin of the larynx, and the common carotid artery on one side was bluntly separated. The vessel was cut using a microscissors to build the model of uncontrollable bleeding. After automatic bleeding for 30 s, the SFM (1.0 g) was applied over the bleeding point and a 100 g compression bag was covered on the wound to provide pressure. The control group was treated with same amount of standard gauze or CELOX-A and same pressure. After bleeding was ceased, the hemostatic time and the blood loss after treatment was recorded. Each group consisted of 6 parallel experiments. The average weight, the standard deviation of rabbits in the SFM, the standard gauze and the CELOX-A groups were 1.90 ± 0.29 Kg, 1.86 ± 0.16 Kg and 1.93 ± 0.09 Kg, respectively.

### 3.5. Hemostatic Mechanism

#### 3.5.1. APTT and PT Test

APTT and PT were measured by using a semiautomatic coagulation analyzer (PUN-2048, Dual Channel Coagulation Analyzer, China). For the APTT test, 2 mg of material, 100 μL of fresh plate-poor plasma (PPP) and 100 μL of APTT reagent were hatched for 5 min at 37 °C. After that, 100 μL of 0.025 M CaCl_2_ in the test cup was measured immediately at 37 °C. The PT test was carried out by blending 2 mg of material and 100 μL of PPP, before incubating for 5 min at 37 °C; then, 100 μL of PT reagent was added to the test cup. The clotting time of APTT and PT were all recorded.

#### 3.5.2. Platelet Adhesion and Morphology

Platelet adhesion was evaluated by a lactate dehydrogenase (LDH) assay. PRP was added to the surface of the materials (2.5 mg) in a centrifuge tube at different incubation times. Nonadherent platelets were washed with PBS for 3 times. Then, adherent platelets were lysed with 1 mL of 1% Triton X-100 at 37 °C for 1 h. Finally, the lactate dehydrogenase activity of platelets was determined by using the LDH Kit (Nanjing Jiancheng Bioengineering Institute, Nanjing, China). A blank served as the control. The number of platelets was calculated according to the standard curve of the platelet count-LDH value. The morphology of the platelets aggregated on the SFM was characterized by SEM. The SFM was immobilized on a glass slide, and a blank glass slide served as a control. The slide was covered with PRP for 30 min, and then the nonadherent platelets were washed away with PBS. The adhered platelets were fixed with 2.5% glutaraldehyde at 4 °C overnight. Subsequently, the samples were dehydrated with 25%, 50%, 75%, and 100% ethanol for 15 min each. Finally, the sample was dried by critical CO_2_, coated with gold and observed by SEM.

#### 3.5.3. Red Blood Cell (RBC) Aggregation and Morphology

After the anticoagulated rabbit blood was centrifuged at 1200× *g* for 10 min, the upper layer of plasma was removed. The lower layer of red blood cells (RBCs) was washed with PBS three times and diluted with PBS solution to obtain a 2% (*v/v*) RBC solution. Then, 150 μL of RBCs was added to the surface of the material (2.5 mg) and incubated at 37 °C for 10 min. After being washed with PBS 3 times to remove the free RBCs, 1 mL of water was added to lyse the RBCs and release hemoglobin from the RBCs. Finally, the absorbance of hemoglobin was measured at 540 nm. The RBC solution was set as the control. The RBC aggregation morphology on the SFM was characterized by SEM. The SFM was immobilized on a glass slide, and a blank glass slide served as the control. A total of 40 μL of RBC solution was added to the surface of the sample and incubated at 37 °C for 30 min. The slide was washed with PBS 5 times to remove the free RBCs and then fixed with 2.5% glutaraldehyde overnight. The SFM was dehydrated in gradient ethanol and dried by critical CO_2_. Finally, the prepared slide was coated with gold and imaged by SEM.

### 3.6. Biocompatibility

#### 3.6.1. Hemolytic Potential of Materials

The hemolytic property measurement was modified according to Yang′s work [39]. Citrated rabbit whole blood (1 mL) was diluted with PBS (5 mL). The sample (10 mg) was then added to PBS (1 mL) to prepare the sample suspension. Next, diluted citrated whole blood (0.1 mL) was added to the sample suspension. After incubation at 37 °C for 1 h, tubes were centrifuged at 150× *g* for 5 min. The absorbance of the supernatant liquid was measured at 540 nm with a microplate reader (SpectraMax ABC plus, Molecular Devices, San Jose, CA, USA). Triton X-100 (0.1%) and PBS were used as the positive and negative controls, respectively. The hemolysis rate was calculated according to the following equation:

(3) $$\mathrm{Hemolysis}\;\mathrm{ratio}(\%)=\frac{\mathrm{sample}\;-\;\mathrm{negative}\;\mathrm{control}}{\mathrm{positive}\;\mathrm{control}\;-\;\mathrm{negative}\;\mathrm{control}}\;\times \;100$$

#### 3.6.2. Cytotoxicity

The cytotoxic effects of samples were estimated on the mouse fibroblast L929 cell line [40]. The material was sterilized by autoclaving at 121 °C for 20 min. L929 cells were incubated in Dulbecco′s modified Eagle′s medium (DMEM) with high glucose (with 100 U/mL penicillin and 100 μg/mL streptomycin) at 37 °C in 5% CO_2_. L929 cells at a density of approximately 1 × 10^4^ cells/well were added to 96-well plates and incubated for 24 h. Then, the medium was replaced with fresh medium, and different doses of sample were added into the wells. The cells were cultured for another 24 h, and cell viability was detected by a Cell Counting Kit-8 (CCK-8, Vazyme Biotech, Nanjing, China).

#### 3.6.3. Acute Systemic Toxicity Test 

Acute systemic toxicity tests were implemented on the basis of the National Standard of the People′s Republic of China (GB/T 14233.2-2005). The test sample was sterilized by an autoclave, and the potential toxin of the sample was extracted by a 9 g/L sterile sodium chloride solution at room temperature for 24 h at a ratio of 0.1 g of sample per 1 mL of sodium chloride solution. The supernatant was filtered through a 0.22 μm membrane filter and injected into the tail veins of SD rats at a dose of 50 mL/kg at a constant speed of less than 0.1 mL/s. The reactions of rats after injection, such as the status, toxicity and mortality, were documented at different times, and the mass of rats was weighed at 72 h. If the reaction of experimental animals was asymptomatic during the observation period, the material was determined to have no acute systemic toxic reaction. 

### 3.7. Statistical Analysis

One subset of data was expressed as the mean ± standard deviation (SD). Statistical significance was evaluated by one-way analysis of variance (ANOVA) between group differences, and a *p* < 0.05 value was considered statistically significant.

## 4. Conclusions

A new discovery of an old material: In this study, we prepared three spongin materials from bath sponge skeletons, which have been widely used in bathing, cleaning, wound caring and so on, for more than 3000 years. All three spongin materials show good hemostatic activity in vitro and in vivo. Among them, the hemostatic activity of the SFM material is particularly outstanding. Through data comparisons, we believe that the hemostatic activity of the SFM is mainly due to three reasons—namely, its hyperabsorbency, high affinity to platelets, RBCs and probably fibrin, and high effective concentration. Overall, results suggest that the SFM will be a fast and safe hemostat for controlling uncontrolled bleeding and exploring the details of their hidden hemostatic mechanisms has in-depth scientific research value. In summary, spongin hemostatic materials not only have good commercial development prospects but also may be suitable for hemostasis in patients with systemic coagulation disorders.

## Data Availability

Not applicable.

## Supplementary Materials

The following are available online at https://www.mdpi.com/article/10.3390/md19040220/s1, Figure S1: The optical microscopy image of *Spongia officinalis* tissue being stained by Sirius red. Figure S2: Standard curve of hydroxyproline concentration to absorbance at 550 nm. Figure S3: Morphologies of SX, SFM and SR. The optical microscopy and SEM images of materials. Figure S4: Photograph of coagulation effect of materials to the PDB. Figure S5: Photograph of the coagulation effect of materials to the DSB. Figure S6: SEM view of platelets aggregation to spongin materials. Figure S7: SEM view of the blood clot formed in DSB by SFM under different conditions. Figure S8: Rabbit RBCs aggregate to spongin materials. Figure S9: SEM view of PDB clot with SFM. Table S1: The weight loss and hydroxyproline contents change in each extraction step. Table S2: Fluid absorption and porosity properties of the different materials. Table S3: Effects of materials on clotting time of rabbit whole blood (sec). Table S4: Effects of materials on clotting time of rabbit PDB (min). Table S5: Effects of materials on clotting time of defibrinated sheep blood (min). Table S6: Coagulation time and blood outflow in tail amputated rats. Table S7: Changes in APTT and PT for rabbit plasma with materials. Table S8: The result of acute systemic toxicity test.
