# Supplementary material for: Rapid Hemostatic Biomaterial from a Natural Bath Sponge Skeleton
Source: Mar Drugs. 2021 Apr 15;19(4):220. doi: 10.3390/md19040220 (PMC8071530; doi:10.3390/md19040220)
Supplement: Supplementary file 1 [file marinedrugs-19-00220-s001.zip › marinedrugs-1186279-supplementary.pdf]

Supporting Information

Rapid Hemostatic Biomaterial from A Natural Bath Sponge Skeleton

Qinghua Wang[‡,1], Jingwei Chen[‡,1], Dexiang Wang[1,2,3], Minghui Shen[4], Huilong Ou[1], Jing Zhao[1,2,3], Ming Chen[1,2,3], Guoliang Yan[5], Jun Chen[*,1,2,3]

[1] Department of Marine Biological Science & Technology, Xiamen University, Xiamen 361102, China; wangqinghua@stu.xmu.edu.cn (Q.W.); cjw1301@163.com (J.C.); dxwang@xmu.edu.cn (D.W.); hlou@xmu.edu.cn (H.O.); sunnyzhaoj@xmu.edu.cn (J.Z.); ming.chen@xmu.edu.cn (M.C.)
[2] State-Province Joint Engineering Laboratory of Marine Bioproducts and Technology, Xiamen University, Xiamen 361102, China
[3] Xiamen City Key Laboratory of Urban Sea Ecological Conservation and Restoration, Xiamen University, Xiamen 361102, China
[4] Hainan Academy of Ocean and Fisheries Sciences, Haikou 570206, China; smh112266@aliyun.com (M.S.)
[5] Basic Medical Department of School of Medicine, Xiamen University, Xiamen 361102, China; guoliangyan@xmu.edu.cn (G.Y.)
[†] Qinghua Wang and Jingwei Chen contributed equally to the article.
[*] Correspondence: chenjun@xmu.edu.cn (J.C.);

Figure S1: The optical microscopy image of *Spongia officinalis* tissue being stained by Sirius red (a) under white light, and (b) under polarized light.

Figure S2: Standard curve of hydroxyproline consentration to absorbance at 550 nm.

Figure S3: Morphology of SX, SFM and SR. The optical microscopy and SEM images of materials..

Figure S4: Photograph of coagulation effect of materials to the PDB.

Figure S5: Photograph of the coagulation effect of materials to the DSB.

Figure S6: SEM view of platelets aggregation to spongin materials.

Figure S7: SEM view of the blood clot formed in DSB by SFM under different details.

Figure S8: Rabbit RBCs aggregate to spongin materials.

Figure S9: SEM view of PDB clot with SFM.

Table S1: The weight loss and hydroxyproline content change in each extraction step.

Table S2: Fluid absorption and porosity properties of the different materials.

Table S3: Effects of materials on clotting time of rabbit whole blood (sec).

Table S4: Effects of materials on clotting time of rabbit PDB (min).

Table S5: Effects of materials on clotting time of defibrinated sheep blood (min).

Table S6: Coagulation time and blood outflow in tail amputated rats.

Table S7: Changes in APTT and PT for rabbit plasma with materials.

Table S8: The result of acute systemic toxicity test.

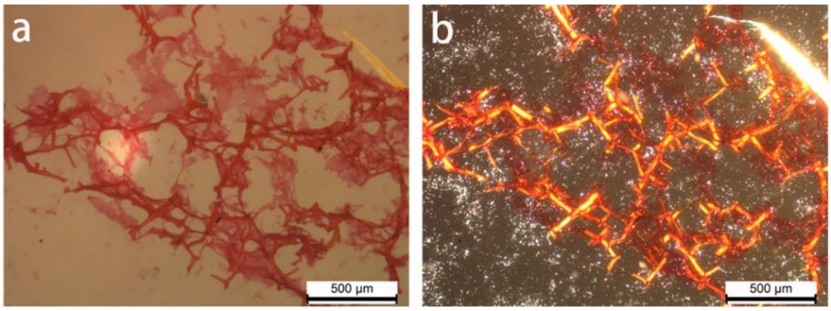

Figure S1. The optical microscopy images of *Spongia officinalis* tissue being stained by Sirius red (a) under white light, and (b) under polarized light.

Due to the unique triple helix structure of collagen, different peptide chains are in the state of parallel polymerization. After collagen was stained by Siriu red, which can stably bind to collagen basic groups, the parallel relationship between dye and collagen resulted in an enhanced birefringence, making collagen molecules of different structures show different colors under polarization microscopy. The fresh tissue of bath sponge *S. officinalis* was sectioned, stained with Siriu red and microscopic observed **(Figure S1**). By comparing microscopy image under white light (**Figure S1a**) and under polarized light (**Figure S1b**), it could be determined that most of the sponge collagen (spongin) was located in the fibre skeleton.

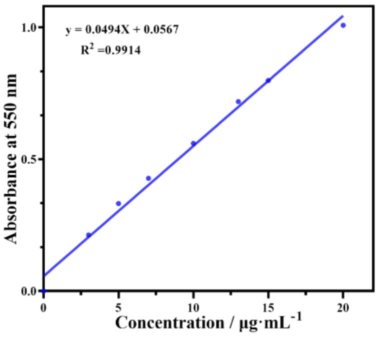

Figure S2. Standard curve of hydroxyproline consentration to absorbance at 550 nm.

The hydroxyproline content of the samples was assayed through the use of hydroxyproline assay kits (A030-2-1, Nanjing Jiancheng Bioengineering Institute, China) by following detail operations.

(a) Establishment of the hydroxyproline standard curve. 5 mg of hydroxyproline (Sinopharm Chemical Reagent Co., Ltd., China) was dissolved in pure water to final 50 mL and obtained 100 μg/ mL mother solution. Then the mother solution was diluted to 3 μg/ mL, 5 μg/mL, 7 μg/ mL, 10 μg/ mL, 13 μg/mL, 15 μg/mL and 20 μg/mL, respectively, and pure water was set as the blank. 1 mL of each solution (or sample hydrolysate, see section (b)) was added with 0.5 mL of chloramine T oxidation solution (from the Kits) and then mixed and standed for 10 min. Following 0.5 mL perchloric acid (from the Kits) was added, mixed and standed for 5 min to neutralize the solution, 0.5 mL pdimethylaminobenzaldehyde chromogenic solution (from the Kits) was added, mixed and then bathed in a 60℃ for 15 min. After cooling and centrifuged at 1500 xg for 10 min, the supernatants were reserved. By zeroed with the blank solution, the absorbances of eath solution at 550 nm were determined. Finally the standard curve of hydroxyproline consentration to absorbance was drawn.

(b) Determination of hydroxyproline content in samples. 20 mg of a sample was

completely immersed into 1 mL of alkaline solution (from the Kits) in a glass tube. The mixure was bathed at 95℃ for 4 h and mixed every 1 h to fully hydrolyze the sample. After the glass tube was cooled to room templature, 0.05 mL methyl orange color indicator (from the Kits) was added and well mixed. The pH of the hydrolysate was adjusted to 6.0-6.8 with indicated color by adding the pH adjusted solution A or B (from the Kits). Then the hydrolysate was fill pure water to a volume of 10 mL in a volumetric flask. 5 mL of the diluted hydrolysate was added with an appropriate amount of activated carbon and shocked for decolorization and then centrifuged at 2000 xg for 15 min and 1 mL of supernatant was taken to determin the hydroxyproline consentration (C_n, μg/mL) by followed the operations descripted in the former section (a). Then the hydroxyproline content (H_n, %) in the sample was caculated as Hn (%) = C_n / 20.

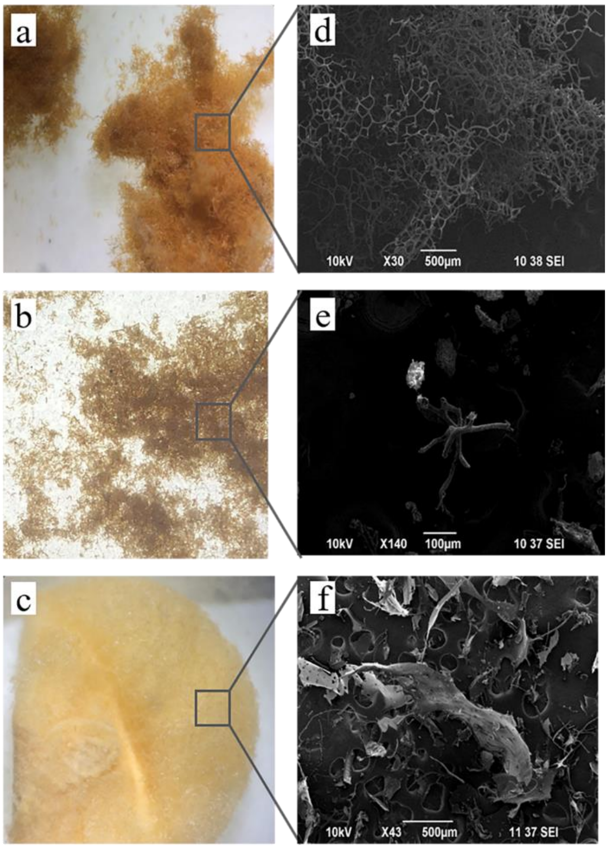

Figure S3. Morphology of SX, SFM and SR. The optical microscopy images of (a) SX, (b) SFM and (c) SR. SEM photographs of (d) SX, (e) SFM and (f) SR.

Yunnan Baiyao, a triditional Chinese hemostatic powder being made from medicinal herbs, could not server as a good control and thus it was not demostrated in the main text. SP, another spongin material, displayed less hemostatic efficacy than SX, SFM and SR and thus it was not demostrated in the main text.

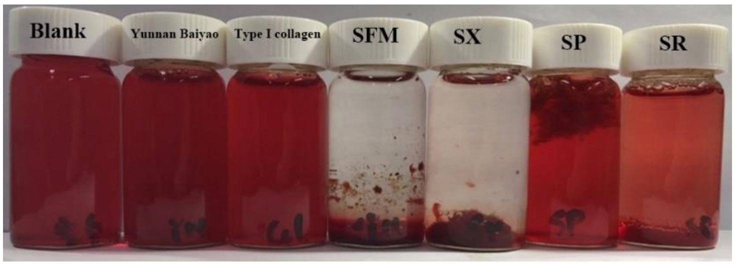

Figure S4. Photograph of coagulation effect of materials to the PDB.

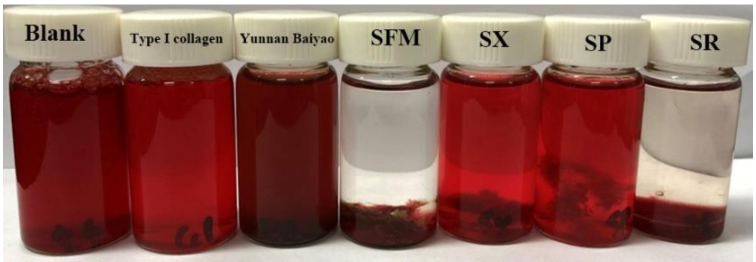

Figure S5. Photograph of the coagulation effect of materials to the DSB.

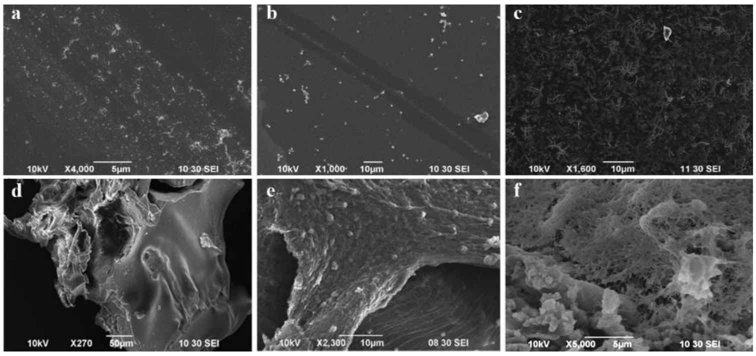

Figure S6 SEM view of platelets aggregation to spongin materials. (a) Original platelet morphology. (b) platelets aggregation to blank slide. (c) platelets aggregation to SR and (d-f) platelet clot around the SFM framework in different details. (d) The integrity of the platelets-SFM clot (e) a tearing surface of the clot and (f) a fishnet structure of one tearing surface.

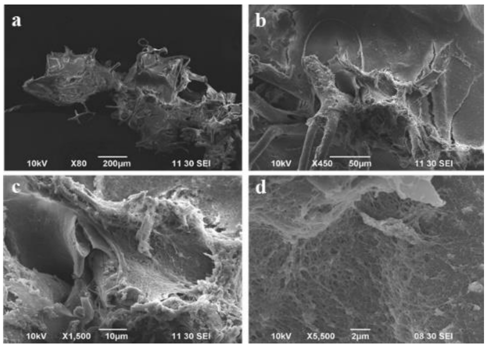

Figure S7. SEM view of the blood clot formed in DSB by SFM under different details.

The DSB-SFM clot sample for SEM observation was prepared as platelets-SFM clot. SFM was immobilized on a glass slide and the slide was covered with DSB for 30 min, and then the nonadherent blood was washed away with PBS. The adhered sample was fixed, dehydrated, dried, coated with gold and observed by SEM as platelets-SFM clot sample.

The integrity, tearing surface and fishnet structure was very similar to the platelet clot around SFM (**Figure S6d-f**), implying the defibrated blood clot was formed by SFM activated.

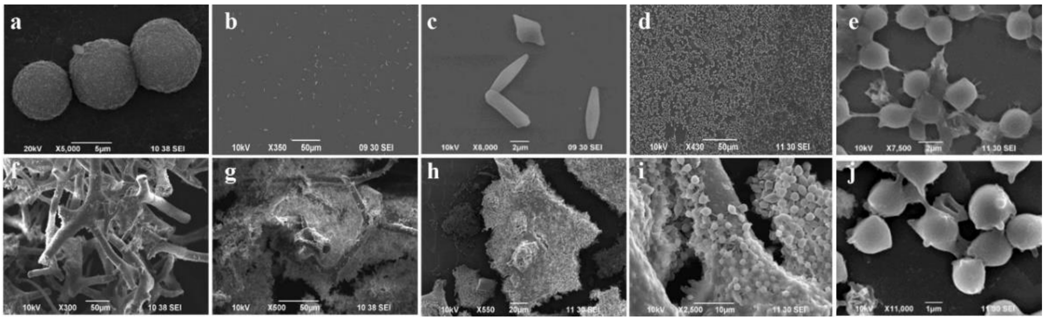

Figure S8. Rabbit RBCs aggregate to spongin materials. (a) Original shape of RBCs. (b, c) RBCs aggregate to blank slide. (d) RBCs aggregate to SR slide. (e) Shape of RBCs after aggregate to SR. (f) SFM before RBCs aggregation. (g, h) RBCs aggregate to SFM. (i, j) Shape of RBCs after aggregate to SFM.

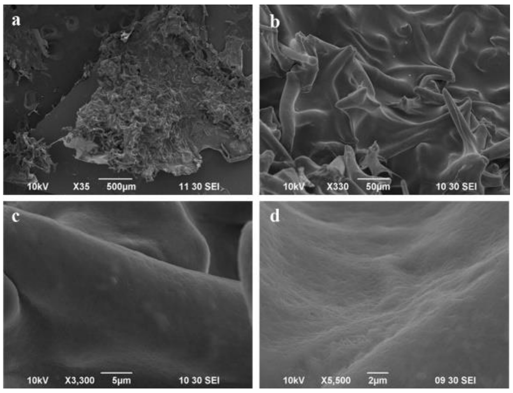

Figure S9. SEM view of PDB clot with SFM. (a) The integrity of the PDB-SFM clot. (b) The clot tightly wrapped on SFM. (c) The clot surface was smooth. (d) In more detail view, a dense network structure and the RBCs underneath it could be observed from the clot surface.

The PDB-SFM clot sample for SEM observation was prepared as platelets-SFM clot and DSB-SFM clot.

Table S1. The weight loss and hydroxyproline content change in each extraction step.

Extraction process	Recovered weight (%)	Hydroxyproline content (%)	Recovered hydroxyproline (%)
1. Initial state	100 ± 0.12	2.27 ± 0.067	0
2. Treatment by 0.8 M HCl for 24 h	65.7 ± 0.84	3.14 ± 0.47	≈ 90.8
3. Treatment by 0.1 M NaOH for 24 h	60.4 ± 1.23	3.40 ± 0.23	≈ 90.4
4. Treatment by 0.1% trypsin for 48 h	36.0 ± 0.8	5.67 ± 0.098	≈ 89.9
4. Treatment by 10% H_2O_2 for 4 h	21.0 ± 0.72	6.03 ± 0.23	≈ 55.8

Table S2. Fluid absorption and porosity properties of the different materials.

Group	SX	SFM	SR	Type I collagen
**Fluid absorption ratio/%**	4050.3 ± 242.6	994.3 ± 84.4	362 ± 31.4	297 ± 22.8
**Porosity/%**	75.40 ± 6.72	90.46 ± 5.56	86.67 ± 7.12	63.82 ± 4.19

Table S3. Effects of materials on clotting time of rabbit whole blood (sec).

Group	1 mg/mL	5 mg/mL	10 mg/mL	13 mg/mL	Control
**Blank**	--	--	--	--	456 ± 20.8
**Type I collagen**	349 ± 17.9	343 ± 60.8	268 ± 10.4	270 ± 20.0	--
**SX**	134 ± 4.0	79 ± 20.8	40 ± 10.0	24 ± 3.6	--
**SFM**	122 ± 2.9	141 ± 44.5	54 ± 5.5	49 ± 8.1	--
**SR**	213 ± 21.9	183 ± 20.2	137 ± 14.4	137 ± 15.3	--

Table S4. Effects of materials on clotting time of rabbit PDB (min).

Group	10 mg/mL	50 mg/mL	100 mg/mL
**Blank**		Uncoagulated	
**Yunnan baiyao**		Uncoagulated	
**Type Ⅰ collagen**		Uncoagulated	
**SX**	40.74 ± 2.91	33.52 ± 0.51	32.37 ± 0.62
**SFM**	25.49 ± 1.11	16.97 ± 1.08	13.73 ± 0.46
**SR**		Uncoagulated	

Table S5. Effects of materials on clotting time of defibrinated sheep blood (min).

Group	10 mg/mL	20 mg/mL	40 mg/mL	60 mg/mL
**Blank**	Uncoagulated			
**Yunnan baiyao**	Uncoagulated			
**Type Ⅰ collagen**	Uncoagulated			
**SX**	Uncoagulated			
**SFM**	No fully coagulated	8.29 ± 0.37	3.18 ± 0.21	2.1 ± 0.14
**SR**	4.92 ± 0.21	3.71 ± 0.12	4.49 ± 0.43	9.23 ± 0.48

Table S6. Coagulation time and blood loss in tail amputated rats.

Group	Clotting Time（s）					Blood loss（mg）				
	**Blank**	**Type I collagen**	**SX**	**SFM**	**SR**	**Blank**	**Type I collagen**	**SX**	**SFM**	**SR**
**1**	970	792	197	83	884	71.3	47.8	17.1	4.4	76.0
**2**	950	619	353	107	577	198.6	8.7	16.5	0	13.3
**3**	1120	878	568	197	801	33.1	26.1	41.6	7.9	19.1
**4**	920	694	296	114	477	94.9	18.1	8.4	5.7	7.3
**5**	941	587	443	145	749	67.4	13.4	27.8	0	24.6
**Mean ±SD**	980 ±80	714 ±108	371 ±127	129 ±39	697 ±149	93.1 ±56.3	22.8 ±13.8	22.3 ±11.4	3.6 ±3.1	28.1 ±24.7

Table S7. Changes in APTT and PT for rabbit plasma with SFM and type I collagen (n=3).

Sample	APTT/s	PT/s
**Blank**	38.2 ± 1.8	20.6 ± 0.65
**SFM**	27 ± 0.35	17.7 ± 0.12
**Type I collagen**	27 ± 5	18.6 ± 0.53

Table S8. The result of acute systemic toxicity test.

Group	SFM		Blank	
	**Initial weight/g**	**72 h weight/g**	**Initial weight/g**	**72 h weight/g**
**1**	260.4	273.7	282.4	294.3
**2**	270.7	277.8	261.5	272.8
**3**	263.4	268.6	272.8	287.2
**4**	251.4	269.2	261.3	274.3
**5**	256.7	270.4	253.7	273.6
**Reaction condition**	Normal		Normal	
**Death**	No death		No death	
